# Supplementary material for: Efficiency of RNA interference is improved by knockdown of dsRNA nucleases in tephritid fruit flies
Source: Open Biol. 2019 Dec 4;9(12):190198. doi: 10.1098/rsob.190198 (PMC6936256; doi:10.1098/rsob.190198)
Supplement: Figure S2. Knockdown of nuclease genes in B. tryoni adults by dsRNA injections [file rsob190198supp1.pdf]

Supplementary information to "Efficiency of RNA interference is improved by knockdown of dsRNA nucleases in tephritid fruit flies" in Open Biology

Alison Tayler, Daniel Heschuk, David Giesbrecht, Jae Yeon Park, and Steve Whyard*
Department of Biological Sciences, University of Manitoba, Winnipeg, MB, R3T 2N2, Canada
DOI: 10.1098/rsob.20160198

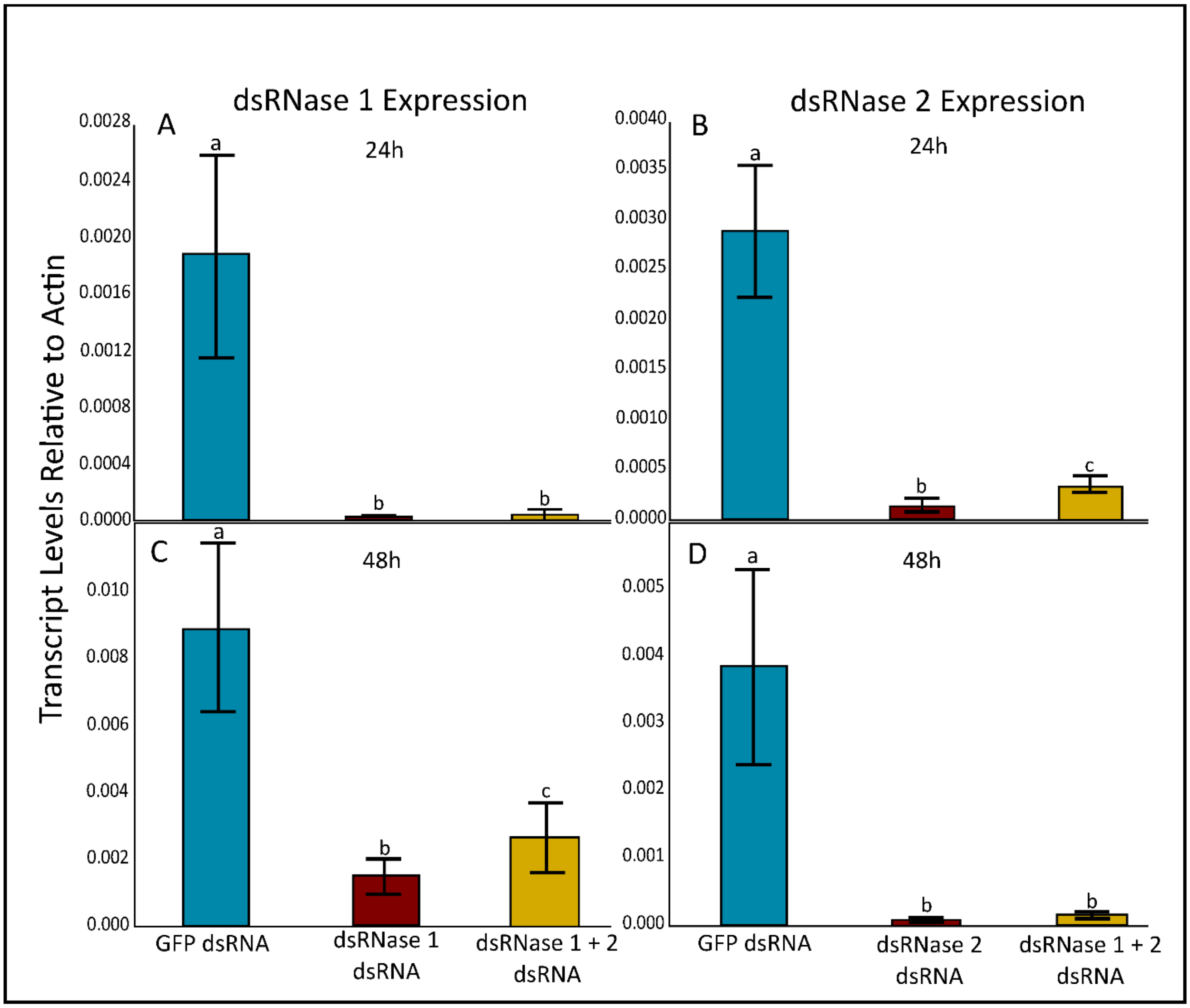

Figure S2. RNAi-mediated knockdown of nuclease genes in *B. tryoni* adults (50:50 males and females) and larvae through haemocoel injections. QRT-PCR analyses were performed on adult whole bodies collected at 24 hours (a,b) and 48 hours (c,d) post-injection. The values represent the means and standard errors for 8 biological replicates. Asterisks indicate significant differences ($p < 0.05$).
