# Supplementary material for: Efficiency of RNA interference is improved by knockdown of dsRNA nucleases in tephritid fruit flies
Source: Open Biol. 2019 Dec 4;9(12):190198. doi: 10.1098/rsob.190198 (PMC6936256; doi:10.1098/rsob.190198)
Supplement: Figure S1. Phylogenetic trees of nuclease genes [file rsob190198supp2.pdf]

Supplementary information to "Efficiency of RNA interference is improved by knockdown of dsRNA nucleases in tephritid fruit flies" in Open Biology

Alison Tayler, Daniel Heschuk, David Giesbrecht, Jae Yeon Park, and Steve Whyard*
Department of Biological Sciences, University of Manitoba, Winnipeg, MB, R3T 2N2, Canada
DOI: 10.1098/rsob.20160198

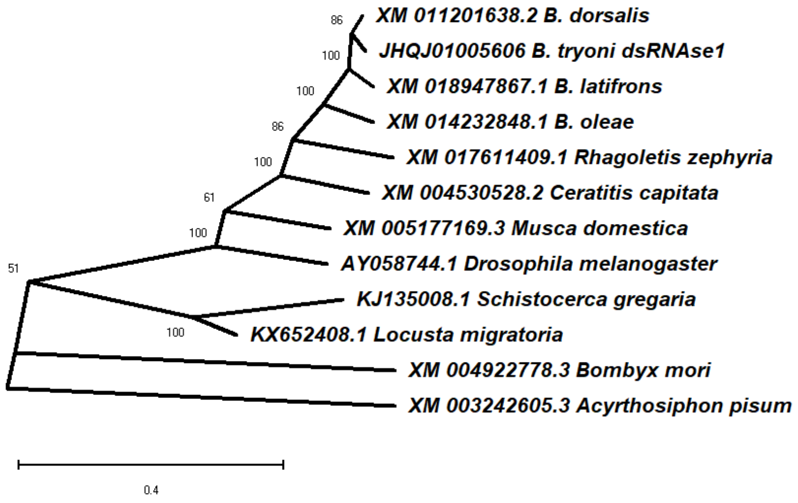

a)

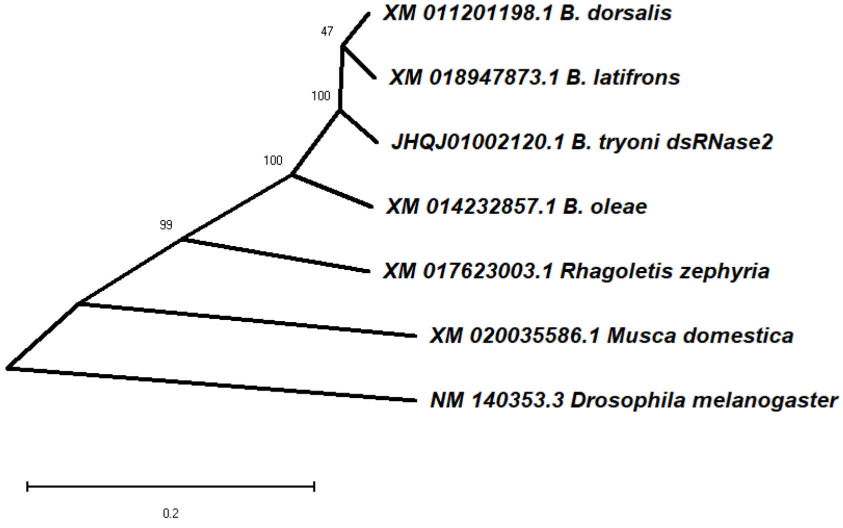

b)

Figure S1. Neighbour-joining phylogenetic trees of a) *dsRNase1* and b) *dsRNase2* with evolutionary distance computed using the maximum composite likelihood method shown on the x-axis. Bootstrap values of 500 replicates are shown at nodes. Alignments were done using CLUSTAL O (1.2.4) and phylogenetic trees constructed and tested using MEGA X.
