# Supplementary material for: Efficiency of RNA interference is improved by knockdown of dsRNA nucleases in tephritid fruit flies
Source: Open Biol. 2019 Dec 4;9(12):190198. doi: 10.1098/rsob.190198 (PMC6936256; doi:10.1098/rsob.190198)
Supplement: Figure S3. dsRNase1 and dsRNase2 knockdown following different dsRNA delivery methods. [file rsob190198supp3.pdf]

Supplementary information to "Efficiency of RNA interference is improved by knockdown of dsRNA nucleases in tephritid fruit flies" in Open Biology

Alison Tayler, Daniel Heschuk, David Giesbrecht, Jae Yeon Park, and Steve Whyard*
Department of Biological Sciences, University of Manitoba, Winnipeg, MB, R3T 2N2, Canada
DOI: 10.1098/rsob.20160198

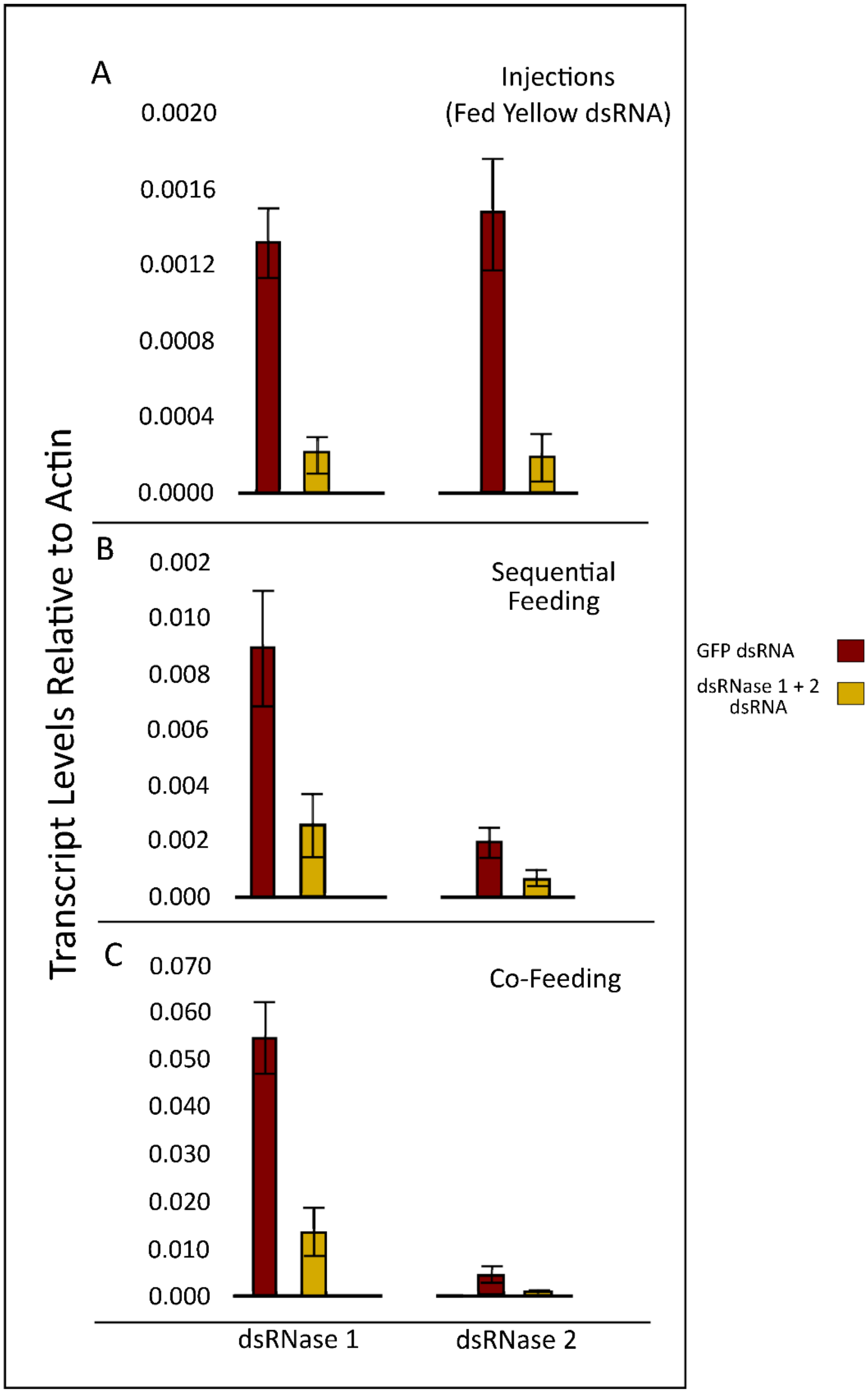

Figure S3. Knockdown of *dsRNase1* and *dsRNase2* following different dsRNA delivery methods. A) DsRNAs were injected into the haemolymph of adults (mixed sexes) and 6 days later, RNA was extracted to assess levels of *dsRNase1* and *dsRNase2* using qRT-PCR. Negative controls were injected with *gfp*-dsRNA, while experimental treatments were injected with a 50:50 mixtures of dsRNAs targeting both nuclease gene transcripts. Values represent the means and standard errors of 8 biological replicates; different letters indicate significant differences (ANOVA with Tukey's *post hoc* test, $p < 0.05$).
