# Supplementary material for: Efficiency of RNA interference is improved by knockdown of dsRNA nucleases in tephritid fruit flies
Source: Open Biol. 2019 Dec 4;9(12):190198. doi: 10.1098/rsob.190198 (PMC6936256; doi:10.1098/rsob.190198)
Supplement: Figure S4. dsRNase1 and dsRNase2 knockdown persistence after larval feeding [file rsob190198supp4.pdf]

Supplementary information to "Efficiency of RNA interference is improved by knockdown of dsRNA nucleases in tephritid fruit flies" in Open Biology

Alison Tayler, Daniel Heschuk, David Giesbrecht, Jae Yeon Park, and Steve Whyard*
Department of Biological Sciences, University of Manitoba, Winnipeg, MB, R3T 2N2, Canada
DOI: 10.1098/rsob.20160198

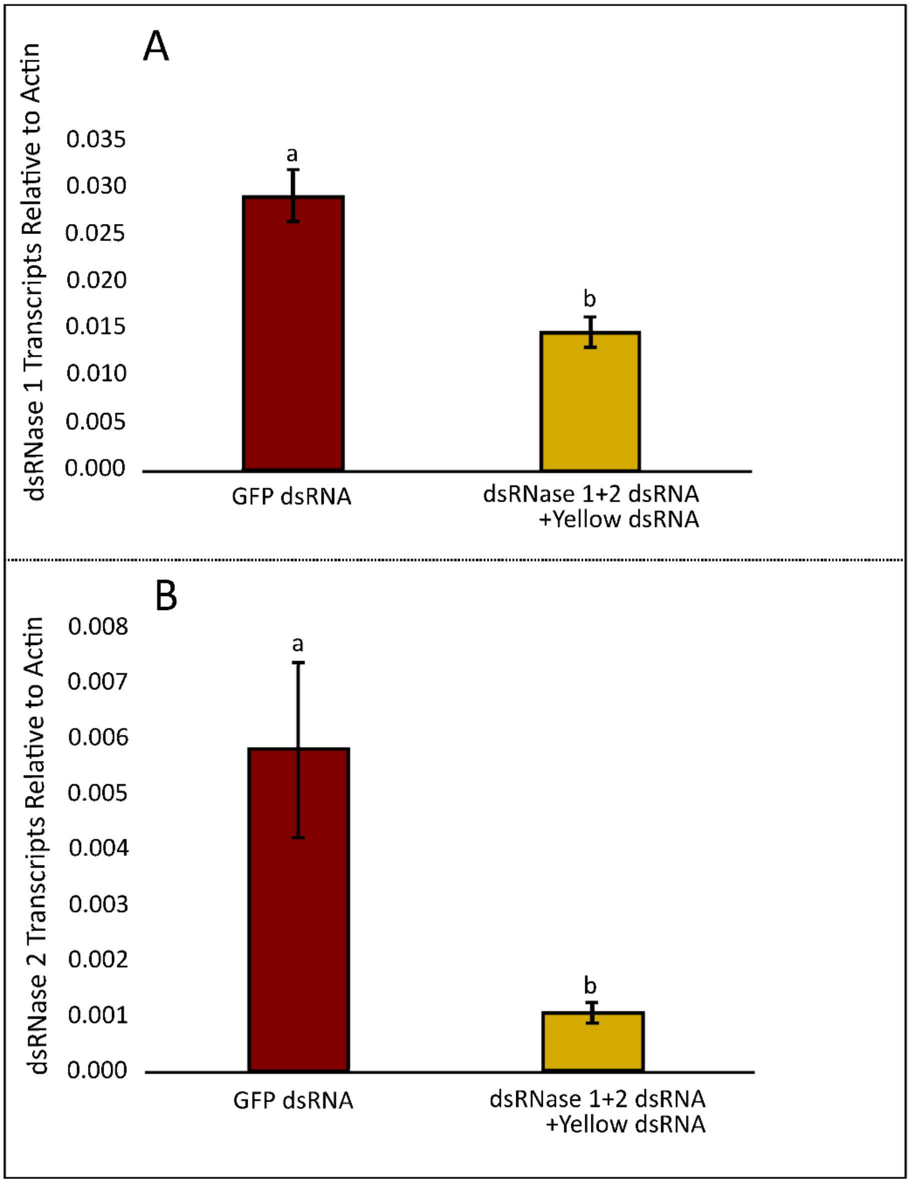

Figure S4. Persistence of knockdown of *dsRNase1* and *dsRNase2* in 5 day old adults (mixed sexes) that had been fed dsRNA during their larval development. Larvae were fed either *gfp*-dsRNA (negative controls) or were fed mixtures of *yellow* dsRNA and dsRNAs targeting both *dsRNase1* and *dsRNase2*. A) Knockdown of *dsRNase1* and B) knockdown of *dsRNase2*. Values represent the means and standard errors of 8 biological replicates; different letters indicate significant differences (ANOVA with Tukey's *post hoc* test, $p < 0.05$).
