# Supplementary material for: Efficiency of RNA interference is improved by knockdown of dsRNA nucleases in tephritid fruit flies
Source: Open Biol. 2019 Dec 4;9(12):190198. doi: 10.1098/rsob.190198 (PMC6936256; doi:10.1098/rsob.190198)
Supplement: Figure S5. Degradation of naked and liposome-encapsulated dsRNA in gut contents [file rsob190198supp5.pdf]

Supplementary information to "Efficiency of RNA interference is improved by knockdown of dsRNA nucleases in tephritid fruit flies" in Open Biology

Alison Tayler, Daniel Heschuk, David Giesbrecht, Jae Yeon Park, and Steve Whyard*
Department of Biological Sciences, University of Manitoba, Winnipeg, MB, R3T 2N2, Canada
DOI: 10.1098/rsob.20160198

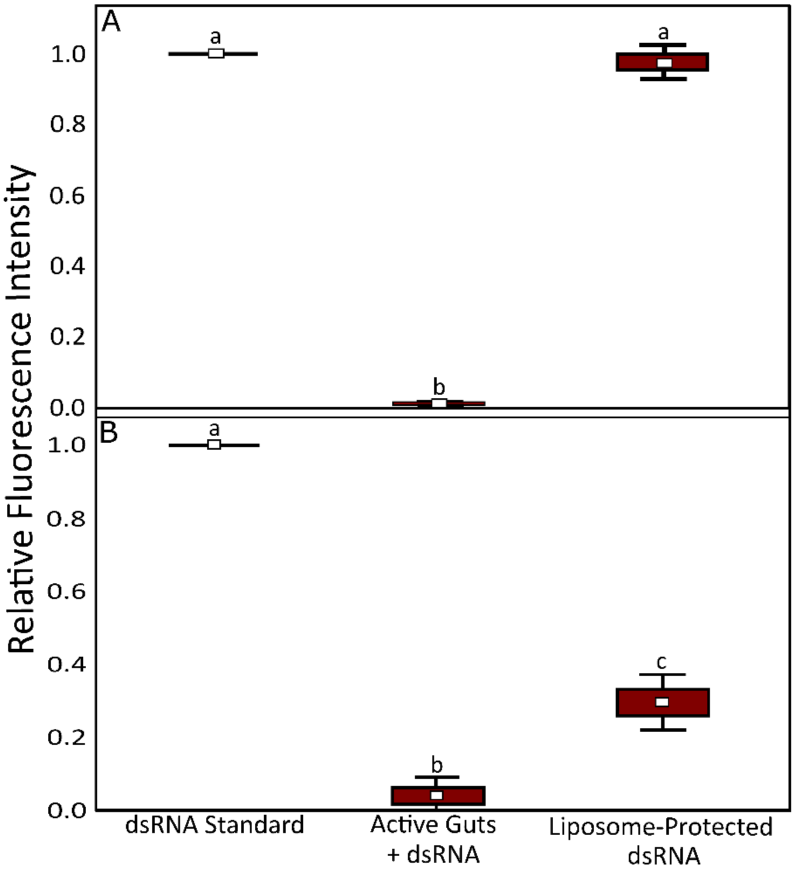

Figure S5. Degradation of naked dsRNA and dsRNA combined with a liposome agent in (mixed sex) adult gut contents measured by band fluorescence intensity following 1.5% gel electrophoresis. A) DsRNA degradation after a 10 minute exposure to gut extracts; non-degraded dsRNA (dsRNA standard) was used as the reference, and dsRNA exposed to gut extracts (active guts + dsRNA) was fully degraded within 10 minutes, while liposome-protected dsRNA remained intact. B) DsRNA degradation after 60 minutes' exposure to gut extracts. Liposomes still provided some protections to the dsRNA.
