# Supplementary material for: Efficiency of RNA interference is improved by knockdown of dsRNA nucleases in tephritid fruit flies
Source: Open Biol. 2019 Dec 4;9(12):190198. doi: 10.1098/rsob.190198 (PMC6936256; doi:10.1098/rsob.190198)
Supplement: Table S1: DNA sequences used to generate dsRNA [file rsob190198supp6.pdf]

Supplementary information to “Efficiency of RNA interference is improved by knockdown of dsRNA nucleases in tephritid fruit flies” in Open Biology

Alison Tayler, Daniel Heschuk, David Giesbrecht, Jae Yeon Park, and Steve Whyard*
Department of Biological Sciences, University of Manitoba, Winnipeg, MB, R3T 2N2, Canada
DOI: 10.1098/rsob.20160198

Table S1: DNA sequences used to generate dsRNA purchased from agroRNA (Seoul, Korea).

Gene name	Sequence
gfp	GAGAAGAACTCTTCACTGGAGTTGGTCCCAGTTCTTGTTGAATTAGATGGCGATGTTAATGGGCAAA AATTCTCTGTCAGTGGAGAGGGTGAAGGTGATGCAACATACGGAAAACTTACCCTTAATTTTATTTGC ACTACTGGGAAGCTACCTGTTCCATGGCCAACACTTGTCACTACTTTCTCTTATGGTGTTCAATGCTTCT CAAGATACCCAGATCATATGAAACAGCATGACTTTTTCAAGAGTGCCATGCCCGAAGGTTATGTACAG GAAAGAACTATATTTTTCAAAGATGAC
gus	CCCTTACGCTGAAGAGATGCTCGACTGGGCAGATGAACATGGCATCGTGGTGATTGATGAAACTGCT GCTGTCGGCTTTAACCTCTCTTTAGGCATTGGTTTCGAAGCGGGCAACAAGCCGAAAGAACTGTACAG CGAAGAGGCAGTCAACGGGGAAACTCAGCAAGCGCACTTACAGGCGATTAAAGAGCTGATAGCGCG TGACAAAAACCACCCAAGCGTGGTGATGTGGAGTATTGCCAACGAACCGGATACCCGTCCGCAAGGT GCACGGGAATATTTCGCGCCACTGGCGGAAGCAACGCGTAAACTCGACCCGACGCGTCCGATCACCT GCGTCAATGTAATGTTCTGCGACGCTCACACCGATACCATCAGCGATCTCTTTGATGTGCTGTGCC
dsRNase1	GTATATCTAGATCGATGGCAAGAGTGTCGATAAACTGTATACTCAAGCCACTCAACTGGCGACCATCA ACGAACATTTGGGCGGTGATGCCAGTAAATACTTTGACTCTGCCAAGAACGTTTACTTAGCACGTGGT CATATGGCCGCCAAAGCTGATTTTGATTATGGTCTCGAACAACGCGCCACTTTCCTCTTCATTAACGCC GCTCCACAGTGGCAGGTCTTCAATGCCGGCAATTGGGCTCGTATCGAAGATGGTGTGCGTGCAAAGG TTTCCTCTGCAGGTTGGTATGTCGATTGCTACACTGGTGTTTACGGAGTGACCACTCTGCCCAACAGC GACGGTGTGCAAACCCCACTCTCGAGTATAC
dsRNase2	TGGTGACGTCGGTAGTTTCATCTGTTACGTCGGTAGTTCCATCAGTTACGTTTATGTATGGAGGTGTG GGGTATGTATCAGCCGTGGATAATGTTGGATATGGTGTGCGGGGTCCATCATTCTCAGTATTGGCAA GTAAGCTTTTCACTACGGTCTCTTTTGAGTGCAGTATGCCTGCCTTCACAACTGCTAATAAGCAAGCTA GCACTAGCACAAGCTTCACTGCTTTTCCGCCCGAATACATTTCGACCGTTCGCGTTAAACTGTTAAAGC CTTGAGAACTATTTGGTATTTAAATAATTGTAGACCATCGAAGTTTGGCACCGAAATCTAATCAAGTTT AGACGAATGTGCCTTCGTAATGGTTGATAACCCATAATCCACCGCAACT
