# Supplementary material for: Efficiency of RNA interference is improved by knockdown of dsRNA nucleases in tephritid fruit flies
Source: Open Biol. 2019 Dec 4;9(12):190198. doi: 10.1098/rsob.190198 (PMC6936256; doi:10.1098/rsob.190198)
Supplement: Table S2. Primers used to amplify gene targets [file rsob190198supp7.pdf]

Supplementary information to "Efficiency of RNA interference is improved by knockdown of dsRNA nucleases in tephritid fruit flies" in Open Biology

Alison Tayler, Daniel Heschuk, David Giesbrecht, Jae Yeon Park, and Steve Whyard*
Department of Biological Sciences, University of Manitoba, Winnipeg, MB, R3T 2N2, Canada
DOI: 10.1098/rsob.20160198

Table S2. Primers used to amplify gene targets in *B. tryoni*.

Gene	Primer	Primer Sequence (5'-3')
Actin	Qfly actin qRT F	CCATGCCATTCTCCGTTTGGATTTG
	Q fly actin qRT R	AGCTGTGGTGGTGAACGAGTAG
dsRNase1	Qfly dsRNase1 RNAi XbaI F	GTATATCTAGATCGATGGCAAGAGTGTCGATAAAC
	Qfly dsRNase1 RNAi XhoI R	GTATACTCGAGAGTGGGGTTTGCACAC
	Qfly dsRNase1 qRT F	CGTTTCGCTCAGCAATATGA
	Qfly dsRNase1 qRT R	AAGGTGATACGGTCCACACC
dsRNase2	Qfly dsRNase2 RNAi XbaI F	GTATATCTAGATGGTGACGTCGGTAGTTTCA
	Qfly dsRNase2 RNAi XhoI R	GTATACTCGAGAGTTGCGGTGGATTATGGG
	Qfly dsRNase2 qRT F	CATTGACATCTGGGAGCGTG
	Qfly dsRNase2 qRT R	CAATTGGGAACGTGTGGAGG
yellow	Qfly yellow RNAi XbaI F	GTATATCTAGACACCCGCTCAAGAACTAAGC
	Qfly yellow RNAi XhoI R	GTATACTCGAGCGTACTCCACATCCACATCG
	Qfly yellow qRT F	CACGTTTGAGTCACGGCGTTCTTT
	Qfly yellow qRT R	CACCAGCAGCGGATTAAGCAATGT
