# Supplementary material for: Efficiency of RNA interference is improved by knockdown of dsRNA nucleases in tephritid fruit flies
Source: Open Biol. 2019 Dec 4;9(12):190198. doi: 10.1098/rsob.190198 (PMC6936256; doi:10.1098/rsob.190198)
Supplement: Table S3. Bactrocera tryoni nuclease sequence similarity [file rsob190198supp8.pdf]

Supplementary information to "Efficiency of RNA interference is improved by knockdown of dsRNA nucleases in tephritid fruit flies" in Open Biology

Alison Tayler, Daniel Heschuk, David Giesbrecht, Jae Yeon Park, and Steve Whyard*
Department of Biological Sciences, University of Manitoba, Winnipeg, MB, R3T 2N2, Canada
DOI: 10.1098/rsob.20160198

Table S3. Percentages of nucleotide similarity between the genome of *Bactrocera tryoni* and nuclease genes in *B. dorsalis* and *Drosophila melanogaster*. Sequence identity analyses were performed using the BLAST program of the National Center for Biotechnology Information (NCBI) (http://blast.ncbi.nlm.nih.gov/Blast.cgi).

Gene	% of nucleotide identity with *B. dorsalis*	% of nucleotide identity with *D. melanogaster*
dsRNase1	95.7 (XM_011201638.2)	70.3 (NM_140819.2)
dsRNase2	94.3 (XM_011201198.1)	67.4 (NM_140353.3)
