# Supplementary material for: Efficiency of RNA interference is improved by knockdown of dsRNA nucleases in tephritid fruit flies
Source: Open Biol. 2019 Dec 4;9(12):190198. doi: 10.1098/rsob.190198 (PMC6936256; doi:10.1098/rsob.190198)
Supplement: Table S4. Bactrocera tryoni yellow sequence similarity [file rsob190198supp9.pdf]

**Supplementary information to "Efficiency of RNA interference is improved by knockdown of dsRNA nucleases in tephritid fruit flies" in Open Biology**

Alison Tayler, Daniel Heschuk, David Giesbrecht, Jae Yeon Park, and Steve Whyard*
Department of Biological Sciences, University of Manitoba, Winnipeg, MB, R3T 2N2, Canada
DOI: 10.1098/rsob.20160198

**Table S4.** Identity matrix of *yellow* genes at the nucleotide level. Sequence identity analyses were performed using the BLAST program of the National Center for Biotechnology Information (NCBI) (http://blast.ncbi.nlm.nih.gov/Blast.cgi).

| **Accession Number and Species Name** | **% Nucleotide Identity** | | | | | | |
|---|---|---|---|---|---|---|---|
| | *Dm* | *Rz* | *Bt* | *Cc* | *Bo* | *Bl* | *Bd* |
| *NM_142031.4_Drosophila_melanogaster (Dm)* | 100 | 53.82 | 48.68 | 53.95 | 52.81 | 55.09 | 54.49 |
| *XM_017625303.1_Rhagoletis_zephyria (Rz)* | 53.82 | 100 | 63.24 | 73.52 | 74.82 | 73.58 | 75.44 |
| *JHQJ01008064.1_B.tryoni (Bt)* | 48.68 | 63.24 | 100 | 63.7 | 88.74 | 91.88 | 93.44 |
| *XM_020859968.1_Ceratitis_capitata (Cc)* | 53.95 | 73.52 | 63.7 | 100 | 73.49 | 72.33 | 74.68 |
| *XM_014235121.1_B.oleae (Bo)* | 52.81 | 74.82 | 88.74 | 73.49 | 100 | 89.81 | 90.75 |
| *XM_018931191.1_B.latifrons (Bl)* | 55.09 | 73.58 | 91.88 | 72.33 | 89.81 | 100 | 95.55 |
| *XM_011210388.2_B.dorsalis (Bd)* | 54.49 | 75.44 | 93.44 | 74.68 | 90.75 | 95.55 | 100 |
